# Consumption of Whole-Grain Bread and Risk of Colorectal Cancer among Norwegian Women (the NOWAC Study)

**DOI:** 10.3390/nu8010040

**Published:** 2016-01-13

**Authors:** Toril Bakken, Tonje Braaten, Anja Olsen, Cecilie Kyrø, Eiliv Lund, Guri Skeie

**Affiliations:** 1 Department of Community Medicine, University of Tromsø–The Arctic University of Norway, P.O. Box 6050 Langnes, N-9037 Tromsø, Norway; tonje.braaten@uit.no (T.B.); eiliv.lund@uit.no (E.L.); guri.skeie@uit.no (G.S.); 2 Danish Cancer Society Research Center, Strandboulevarden 49, DK-2100 Copenhagen Ø, Denmark; anja@cancer.dk (A.O.); ceciliek@cancer.dk (C.K.)

**Keywords:** colorectal cancer, proximal colon, whole-grain, bread, cohort, prospective, women, Norway

## Abstract

There is evidence that consumption of foods containing dietary fiber decreases the risk of colorectal cancer (CRC). Whole grains contain dietary fiber, as well as a range of micronutrients and bioactive compounds, but the association between the consumption of whole grains and the risk of CRC remains less studied. The aim of the present study was to investigate the association between whole-grain bread consumption and CRC incidence among Norwegian women, using data from a prospective cohort study (the Norwegian Women and Cancer Study). Dietary intake was estimated from the food-frequency questionnaires of 78,254 women in the cohort (median age: 55 years), and these women were then followed up for CRC incidence. During the 9 years of median follow-up, 795 women were diagnosed with CRC (316 proximal, 193 distal, 218 rectal). Associations between whole-grain bread consumption and the risk of CRC (including colorectal subsites) were investigated using Cox proportional hazards regression models. When compared to the low consumption group, the hazard ratio for CRC was 0.89 (95% confidence interval (CI): 0.72–1.09) for the high consumption group and 0.86 (95% CI: 0.72–1.02) for the medium consumption group in a multivariable model. Overall, no association between whole-grain bread consumption and CRC was found.

## 1. Introduction

Colorectal cancer (CRC) is the second most common cancer in women and the third most common in men worldwide [1]. There is large geographical variation in CRC incidence across the world [1], with high incidence related to economic development [2]. In Norway, the incidence of colon cancer among women has almost tripled the last 55 years [3], and CRC incidence among women is one of the highest in the world [4].

Diet is believed to play a role in the development of CRC [5]. According to the World Cancer Research Fund/American Institute for Cancer Research, there is convincing evidence that intake of foods containing dietary fiber decreases the risk of CRC [5], and studies have suggested that dietary fiber from cereal sources is especially associated with this lower risk [6,7]. Whole grains are an important source of dietary fiber, but contrary to refined grains, whole grains contain more micronutrients, as well as a range of bioactive components [8,9,10]. While the association between dietary fiber intake and the risk of CRC has been investigated in many cohorts [5], the association with consumption of whole grains has been less studied. A meta-analysis [6] and a systematic review [11] indicated that there may be an inverse association between consumption of whole grains and risk of CRC. However, findings are not consistent [12,13,14,15,16,17,18,19,20].

Bread is a staple food in Norway, accounting for 21% of energy intake [21,22,23], and bread is the most important source of whole grains in Norway [24,25]. The aim of the present study was to investigate the association between whole-grain bread consumption and CRC incidence among Norwegian women.

## 2. Methods

### 2.1. The NOWAC Study, Exclusion Criteria and Study Sample

The Norwegian Women and Cancer (NOWAC) Study is a nationwide prospective cohort study consisting of approximately 172,000 women. The study has been enrolling women for 15 years, with the first wave of enrollment taking place in 1991. All study participants complete a mailed questionnaire at enrollment, and follow-up questionnaires are sent approximately every 6th year after enrollment. NOWAC participants who answered an enrollment questionnaire, first follow-up questionnaire, or second follow-up questionnaire between 2002 and 2006 (*n* = 90,592) were eligible for inclusion in the present analysis. The reason for including enrollment questionnaire, first and second follow-up questionnaires, is that these questionnaires had the same questions about bread consumption (the bread questions in the NOWAC questionnaire have varied over time). For each participant, information from only one questionnaire was used in the analyses.

We excluded women diagnosed with any invasive cancer prior to the start of follow-up (*n* = 4887), as well as those who had follow-up <1 year (*n* = 649). We further excluded those with International Classification of Diseases for Oncology (ICD-O) tumor behavior code 6 (malignant, metastatic site) (*n* = 1) or ICD-O code 9 (malignant, uncertain whether primary or metastatic site) (*n* = 16) and those with a daily energy intake <2.5 MJ (*n* = 1061) or >15.0 MJ (*n* = 153). Women with missing information on any of the following variables were also excluded: all three questions on bread consumption (whole-grain bread, partly refined bread and refined bread) (*n* = 1124), self-reported weight (*n* = 2582), self-reported height (*n* = 279), smoking status (*n* = 1380) and smoking history (*n* = 198). We further excluded those with body mass index (BMI) <13.0 kg·m^−2^ (*n* = 3) or >60.0 kg·m^−2^ (*n* = 4). Finally, one woman who returned the questionnaire in 2007 was excluded. Thus a total of 78,254 women, which included three questionnaire subcohorts (31% who answered the enrollment questionnaire, 30% who answered the first follow-up questionnaire and 39% who answered the second follow-up questionnaire) were included in the present analyses.

The NOWAC Study has been approved by the Regional Committee for Medical Research Ethics and the Norwegian Data Inspectorate and informed consent has been obtained from the participants. The NOWAC Study and corresponding validation studies have been described previously [26,27,28,29,30,31].

### 2.2. Assessment of Dietary and Non-Dietary Exposures

Information on age and municipality of residence was extracted through linkage to the population register (Statistics Norway). Information on education was taken from the enrollment questionnaire for the entire study sample. All other exposure data used in the analysis were taken from the questionnaire the participants completed during the study period, *i.e.*, the enrollment questionnaire, first follow-up questionnaire, or second follow-up questionnaire.

BMI was calculated from self-reported weight and height, both of which have been validated [31]. Information on smoking status and smoking history was combined into one variable of smoking duration. Physical activity level (global score including physical activity at home, at work, exercise, walking and such) was reported according to a 10-point scale, which was found to be valid to rank physical activity level among the women in the study [26].

All of the considered questionnaires included a semi-quantitative food frequency questionnaire (FFQ), which covered food items commonly eaten in Norway, but not the total diet. A validation study found that the FFQ’s ability to rank NOWAC participants was good for foods eaten frequently [27]. The Spearman’s rank correlation coefficient between the FFQ and the four repeated 24-h dietary recalls (24HDRs) was 0.49 for “Bread and cereals” [27]. For dietary fiber, calcium and energy the Spearman’s rank correlation coefficient between the FFQ and the 24HDRs was 0.48, 0.50 and 0.30, respectively [27]. A test-retest reproducibility study found that the level of reproducibility observed for the FFQ used was within the range reported for similar instruments [30].

Participants reported their average consumption of foods during the last year in the FFQ by ticking fixed frequencies. Knowledge about Norwegian food habits was the basis when the frequency options, including the number of frequency options, were chosen. Participants reported the portion size of a food consumed in natural units (for example banana), household units (for example tablespoons) or deciliters. We multiplied the mean of the frequency option by the portion size to calculate the consumption of each food (in g). For the uppermost category of a food, the lowest value in the frequency option was used (for example, a frequency option of “3+”, was categorized as intake of “3” in our analyses). The Norwegian Weight and Measurement Table [32] was used in these calculations. Daily intake of energy and nutrients was computed based on the Norwegian Food Composition Table [33]. Missing frequencies were treated as no consumption, and missing portion sizes as the smallest portion unit in the questionnaire. Daily intakes of foods, nutrients, and energy were calculated for each participant using a program developed at the Institute of Community Medicine, UiT The Arctic University of Norway, for SAS software (SAS Institute Inc., Cary, NC, USA).

Participants reported their consumption of bread in slices of different bread types (whole-grain bread, partly refined bread, or refined bread). Moreover, six different frequency options were included (zero/seldom, 1–4 slices·week^−1^, 5–7 slices·week^−1^, 2–3 slices·day^−1^, 4–5 slices·day^−1^ and ≥6 slices·day^−1^). “Seldom” covers more than zero consumption, but <1 slice week^−1^. Study participants who reported a “zero/seldom” consumption of bread, were categorized as consuming no bread in our analyses. One slice of whole-grain bread was estimated to weigh 40 g and one slice of partly refined bread to weigh 30 g [32]. Consumption of red meat included steak, chops and roast meat (beef, pork, mutton), and processed meat included sausages, meatballs, hamburgers, sandwich meats, and liver pate.

### 2.3. Vital Status and Classification of CRC Cases

The unique national identification number given to all Norwegian citizens was used to link NOWAC participants to the population register (Statistics Norway) for vital status (alive, emigrated, dead), and to the Cancer Registry of Norway for cancer incidence.

CRC cases were classified according to the International Classification of Diseases, Seventh Revision (ICD-7) by the Cancer Registry of Norway and further classified according to anatomic subsite: CRC (ICD-7 code 153.0–154.0); colon cancer (ICD-7 code 153.0–153.9); proximal colon cancer (ICD-7 code 153.0–153.1: cecum, ascending and transverse colon including hepatic and splenic flexures); distal colon cancer (ICD-7 code 153.2–153.3: descending and sigmoid colon); and rectal cancer (ICD-7 code 154.0). Cancers of the colon with codes 153.4 (recto-sigmoid junction), 153.6 (appendix), and 153.9 (unspecified location) were only included in the analyses for total colon cancer. Anal canal tumors (ICD-7 code 154.1) were not included in the analyses. Moreover, only cases with ICD-O tumor behavior code 3 (malignant, primary cite) were classified as CRC.

### 2.4. Statistical Analysis

Descriptive characteristics of the participants at the start of follow-up are presented as median values with 5th and 95th percentiles or as percentages. We used Kruskal-Wallis equality of populations rank test and Spearman’s rank correlation coefficient for examining differences in the distribution of baseline characteristics between the whole-grain bread consumption groups. The associations between whole-grain bread consumption and incidence of total CRC, total colon, proximal colon, distal colon, and rectal cancer were investigated in Cox proportional hazards regression models with age as the time scale. All models were stratified by the questionnaire subcohorts. Hazard ratios (HRs) with 95% confidence intervals (CIs) were calculated.

Start of follow-up was set as the age of the woman at the time the questionnaire was returned. Person-years were calculated from the start of follow-up to age at diagnosis of any cancer (except non-melanoma skin cancer), emigration, death, or the end of follow up (31 December 2013), whichever occurred first.

A Cox proportional hazards regression model (full model) was established with whole-grain bread consumption as the exposure and adjusted for predefined potential confounders for CRC selected according to a literature review (BMI, hormone replacement therapy, smoking, alcohol consumption, red meat consumption, processed meat consumption, fiber from foods other than whole-grain bread, and calcium from food). Two additional models are presented: an age-adjusted model (with age as the timescale) and an energy-adjusted full model (adjusting for everything in the full model plus energy).

Adjusting for physical activity level (*n* = 72,175) did not change the HRs for total CRC and the colorectal subsites in the age-adjusted and the full model, respectively. Due to this, the missing information on physical activity level (*n* = 6079), and the moderate power in the study, physical activity level was not included in the full model.

Whole-grain bread consumption was modelled as a categorical variable; the groups were classified as zero/seldom (0 g·day^−1^), low (14/34 g·day^−1^), medium (100 g·day^−1^) and high (180/240 g·day^−1^). A zero/seldom consumption group was created since whole-grain bread consumption is common in Norway [22] and therefore those reporting zero/seldom whole-grain bread consumption may differ from the rest of the population, for instance they might have potential health related reasons for not eating whole-grain bread.

The following variables were modelled categorically: hormone replacement therapy (never/former, current); smoking (never, smoking < 30 years, smoking ≥ 30 years); physical activity level (on a 10-point scale: 1–3, low; 4–7, moderate; 8–10, high); duration of education (<10 years, 10–12 years, >12 years); area of residence (East, Oslo, South-East, West, Middle, North); an indicator variable for period of birth (year of birth: ˂1944; 1944–1953; ˃1953); partly refined bread consumption (0, 11/26, 75/135, 180 g·day^−1^); and potato consumption (0/22, 50/63, 126, 189/252 g·day^−1^). Furthermore, the following variables were modelled continuously: BMI (kg·m^−^^2^); red meat consumption (g·day^−1^); processed meat consumption (g·day^−1^); fiber from foods other than whole-grain bread (g·day^−1^); calcium from food (mg·day^−1^); alcohol consumption (g·day^−1^); energy intake (kJ·day^−1^); height (cm); folate from foods other than whole-grain bread (µg·day^−1^); fruit consumption (g·day^−1^); and vegetable consumption (g·day^−1^).

The three predefined models were used when investigating the associations between whole-grain bread consumption and incidence of total CRC and of cancer at colorectal subsites. The low whole-grain bread consumption group was used as a reference category in the analyses, since those in the zero/seldom group may differ from the rest of the population and there were few colorectal cases in the zero/seldom group.

Additional analyses were performed to examine the robustness of the associations for total CRC and the colorectal subsites. First, we ran analyses using the full model less one variable (BMI, hormone replacement therapy, smoking, alcohol consumption, red meat consumption, processed meat consumption, fiber from foods other than whole-grain bread, and calcium from food) to determine if the removal of this variable affected the results. We also performed separate analyses using the full model plus one variable (height, area of residence, period of birth, partly refined bread and folate from foods other than whole-grain bread) to determine if the addition of this variable affected the results. Then we examined the effect of using fruit, vegetables, and potatoes instead of fiber from foods other than whole-grain bread in the full model. We also investigated whether adding education (excluding women with missing information on education (*n* = 3774), women with <7 years of education (*n* = 289) and >29 years of education (*n* = 26)) affected the associations. Finally, we excluded all CRC cases with follow-up ≤1 year to eliminate the influence of pre-existing disease on the estimates of the associations for total CRC and the colorectal subsites.

We observed only minor effects on the estimated regression coefficients in the full model for total CRC and the colorectal subsites when the continuous variables were changed to categorical variables. Due to this and moderate power in the study, these variables were modelled continuously. Correlations between the variables included in the Cox proportional hazards regression models were assessed by Spearman’s correlation coefficient and variance inflation factor. The proportional-hazards assumption was examined using Schoenfeld residuals and by a log-log survival plot. Possible predefined interaction effects (whole-grain bread *versus* red and processed meat, respectively [34]) were investigated in the full model for total CRC and the colorectal subsites using the likelihood ratio test comparing the full model and the full model including a product term. To test for linear trend in risk, we made a continuous variable by calculating the average consumption of whole-grain bread within each whole-grain bread group and including this continuous variable in the Cox proportional hazards regression models.

Analyses were carried out using STATA (StataCorp, College Station, TX, USA) version 14.0.

## 3. Results

During the 717,482 person-years of observation, 795, 577 (73%) and 218 (27%) incident cases of CRC (total) and colon (total) and rectal cancer, respectively, were reported in the study sample. Among the colon cancers, 316 (55%) were proximal, and 193 (33%) were distal; the rest were cancer of the recto-sigmoid junction/appendix/unspecified location. Median follow-up was 9 (range 1–11) years, and the median age at CRC diagnosis was 65 (range 48–86) years.

Those eating more whole-grain bread reported less smoking and a higher physical activity level (Table 1). The median age at start of follow-up was equal in all consumption groups (55 years, range across all consumption groups 46–76 years) and the median BMI was similar in all consumption groups.

**Table 1:** Baseline characteristics (2002–2006) of the study sample (Norwegian Women and Cancer Study), *n* = 78,254.

Variables	Whole-Grain Bread Consumption Group ^1^				*p* ^2^
	Zero/Seldom 0 g·Day^−1^ *n* = 8095	Low 14 or 34 g·Day^−1^ *n* = 17,396	Medium 100 g·Day^−1^ *n* = 33,994	High 180 or 240 g·Day^−1^ *n* = 18,769	
	**Median (5th–95th Percentile)**				
Age at start of follow-up (years)	55 (47–70)	55 (47–68)	55 (48–69)	55 (47–67)	˂0.001
BMI (kg·m^−2^)	25 (20–33)	25 (20–33)	25 (20–33)	24 (20–33)	˂0.001
	**%**				
Current HRT use	21	21	22	20	˂0.02
Smoking:					˂0.001
Never	33	33	37	38	
<30 years	33	38	37	35	
≥30 years	34	29	26	26	
Physical activity level: ^3^					˂0.001
Low	18	13	10	9	
Moderate	69	70	72	71	
High	13	17	17	20	
Education: ^4^					˂0.001
<10 years	31	21	21	22	
10–12 years	36	36	35	34	
>12 years	33	43	44	45	
	**Median (5th–95th Percentile)**				
Partly refined bread (g·day^−1^)	75 (0–135)	0 (0–135)	0 (0–75)	0 (0–26)	˂0.001
Refined bread (g·day^−1^)	0 (0–21)	0 (0–9)	0 (0–9)	0 (0–9)	˂0.001
Crisp bread (g·day^−1^)	4 (0–55)	4 (0–55)	4 (0–31)	4 (0–31)	˂0.001
Breakfast cereals (g·day^−1^)	0 (0–72)	0 (0–72)	0 (0–72)	0 (0–51)	˂0.001
Fruit (g·day^−1^)	150 (14–481)	192 (26–506)	208 (33–506)	208 (33–508)	˂0.001
Vegetables (g·day^−1^)	111 (24–313)	148 (40–364)	148 (43–350)	150 (44–352)	˂0.001
Potatoes (g·day^−1^)	126 (0–189)	63 (0–189)	63 (22–189)	126 (22–189)	˂0.001
Red meat (g·day^−1^)	14 (0–41)	13 (0–38)	13 (0–36)	13 (0–37)	˂0.001
Processed meat (g·day^−1^)	30 (4–74)	24 (4–65)	26 (4–63)	31 (4–74)	˂0.001
Fiber other than whole-grain bread (g·day^−1^)	16 (8–27)	16 (8–27)	15 (7–26)	15 (8–26)	˂0.001
Calcium from food (mg·day^−1^)	613 (270–1250)	580 (291–1155)	650 (333–1213)	761 (364–1367)	˂0.001
Folate other than whole-grain bread (µg·day^−1^)	175 (97–282)	172 (92–287)	174 (96–288)	180 (100–294)	˂0.001
Alcohol (g·day^−1^)	2 (0–13)	2 (0–13)	2 (0–13)	2 (0–12)	˂0.001
Energy intake (kJ·day^−1^)	6325 (3614–9761)	6005 (3514–9381)	6811 (4476–9974)	8176 (5704–11,612)	˂0.001

^1^: Consumption of six or more slices of bread day^−1^ was categorized as consuming six slices (240 g) day^−1^ in our analyses; ^2^: Kruskal-Wallis equality of populations rank test or Spearman’s rank correlation coefficient; ^3^: *n* = 72,175; ^4^: *n* = 74,165; BMI: body mass index, HRT = hormone replacement therapy.

The range in whole-grain bread consumption was 0–240 g·day^−1^. The consumption of crisp bread and breakfast cereals, two other foods which may contain whole grains and therefore may confound the results of our analyses, were low: median consumption was 4 (range 0–74) g·day^−1^ for crisp bread and zero/seldom (range 0–72) g·day^−1^ for breakfast cereals in all the whole-grain bread consumption groups.

The median consumption of whole-grain bread was the same in the total sample and among CRC cases (Table 2). Also, the median consumption of partly refined and refined bread did not differ among the total sample and the CRC cases. The median BMI was the same among the total sample and the CRC cases. At the start of follow-up, women developing CRC were older and fewer reported being never smokers than the total sample.

When investigating the associations between whole-grain bread consumption and incidence of total CRC and cancer at the colorectal subsites in the three different models (age-adjusted, full model, and energy-adjusted full model), overall no association between whole-grain bread consumption and CRC was found (Table 3). When comparing the high and the medium consumption groups with the low consumption group, the HRs for total CRC were 0.89 (95% CI: 0.72–1.09) and 0.86 (95% CI: 0.72–1.02), respectively, in the full model. When performing the same comparisons for total colon cancer in the full model, the HRs were 0.91 (95% CI: 0.71–1.16) and 0.81 (95% CI: 0.66–1.00), respectively. For proximal colon cancer, the HRs in the full model were 0.79 (95% CI: 0.57–1.10) and 0.75 (95% CI: 0.57–0.99), respectively, when comparing the high consumption group and the medium consumption group with the low consumption group. When performing the same comparisons for distal colon cancer in the full model, the HRs were 0.96 (95% CI: 0.64–1.45) and 0.73 (95% CI: 0.51–1.05), respectively. Risk estimates for women in the zero/seldom consumption group were below 1.00 for both proximal and distal colon cancer in all three models. However, none of these estimates were statistically significant at the 5% level. No association was found between whole-grain bread consumption and rectal cancer. Additional adjustment for energy intake did not substantially change the HRs in the full model for total CRC or any of the colorectal subsites. No linear trend was found for total CRC or any of the subsites.

**Table 2:** Baseline characteristics (2002–2006) of the study sample and colorectal cancer (CRC) cases by total CRC and colorectal subsites (Norwegian Women and Cancer Study), *n* = 78,254.

Variables	Total Sample *n* = 78,254	Total CRC *n* = 795	Total Colon *n* = 577	Proximal Colon *n* = 316	Distal Colon *n* = 193	Rectal *n* = 218
	**Median (5th–95th Percentile)**					
Age at start of follow-up (years)	55 (47–68)	59 (49–73)	59 (50–74)	60 (50–74)	58 (49–73)	57 (49–71)
BMI (kg·m^−2^)	25 (20–33)	25 (20–33)	25 (20–34)	25 (20–34)	25 (21–33)	25 (20–33)
	%					
Current HRT use	21	22	21	20	23	26
Smoking:						
Never	36	32	31	32	31	33
<30 years	36	37	36	33	40	40
≥30 years	28	31	33	35	29	27
Physical activity level: ^1^						
Low	11	13	13	12	16	12
Moderate	71	72	72	75	67	72
High	17	15	15	13	17	16
Education: ^2^						
<10 years	22	33	36	39	33	28
10–12 years	35	36	36	36	35	34
>12 years	43	31	28	25	32	39
	**Median (5th–95th Percentile)**					
Whole-grain bread (g·day^−1^)	100 (0–180)	100 (0–180)	100 (0–180)	100 (0–180)	100 (0–180)	100 (0–180)
Partly refined bread (g·day^−1^)	0 (0–135)	0 (0–75)	0 (0–75)	0 (0–75)	0 (0–75)	0 (0–135)
Refined bread (g·day^−1^)	0 (0–9)	0 (0–9)	0 (0–9)	0 (0–9)	0 (0–9)	0 (0–9)
Fruit (g·day^−1^)	201 (29–500)	185 (23–486)	191 (23–486)	192 (19–472)	181 (33–481)	182 (26–486)
Vegetables (g·day^−1^)	145 (40–351)	131 (33–360)	130 (33–346)	122 (30–366)	135 (39–307)	134 (31–366)
Potatoes (g·day^−1^)	63 (0–189)	126 (22–189)	126 (22–189)	126 (22–189)	126 (22–189)	126 (0–189)
Red meat (g·day^−1^)	13 (0–37)	13 (0–41)	13 (0–42)	13 (0–43)	13 (0–36)	13 (0–41)
Processed meat (g·day^−1^)	26 (4–68)	26 (4–67)	26 (4–66)	26 (4–66)	25 (4–66)	26 (2–74)
Fiber other than whole-grain bread (g·day^−1^)	16 (8–27)	15 (7–26)	15 (7–26)	15 (7–27)	15 (7–25)	15 (8–26)
Calcium from food (mg·day^−1^)	653 (318–1254)	627 (306–1195)	627 (306–1175)	613 (300–1151)	637 (332–1219)	626 (287–1264)
Folate other than whole-grain bread (µg·day^−1^)	175 (96–289)	169 (89–286)	167 (89–284)	166 (87–294)	167 (88–261)	174 (89–295)
Alcohol (g·day^−1^)	2 (0–13)	2 (0–12)	2 (0–12)	1 (0–11)	2 (0–12)	2 (0–11)
Energy intake (kJ·day^−1^)	6931 (4135–10,468)	6690 (3937–10,341)	6670 (3894–10,439)	6678 (3758–10,540)	6540 (3867–9793)	6767 (3947–10,223)

^1^: *n* = 72,175; ^2^: *n* = 74,165; BMI: body mass index; HRT = hormone replacement therapy.

**Table 3:** Hazard ratios and 95% confidence intervals for the risk of total colorectal cancer (CRC) and colorectal subsites according to whole-grain bread consumption in the Norwegian Women and Cancer Study, *n* = 78,254.

	Whole-Grain Bread Consumption Group ^1^				*p* For Linear Trend
	Zero/Seldom	Low	Medium	High	
Person-years	74,397	159,394	312,445	171,246	
	**Total CRC**				
Cases	87	196	336	176	
Age-adjusted model ^2^	0.89 (0.69–1.14)	1 (ref)	0.83 (0.70–0.99)	0.85 (0.69–1.04)	0.17
Full model ^3^	0.87 (0.68–1.12)	1 (ref)	0.86 (0.72–1.02)	0.89 (0.72–1.09)	0.44
Full model + energy ^4^	0.87 (0.67–1.12)	1 (ref)	0.84 (0.70–1.01)	0.85 (0.66–1.09)	0.34
	**Total Colon**				
Cases	61	146	238	132	
Age-adjusted model	0.82 (0.61–1.11)	1 (ref)	0.79 (0.64–0.97)	0.86 (0.68–1.08)	0.37
Full model	0.81 (0.60–1.09)	1 (ref)	0.81 (0.66–1.00)	0.91 (0.71–1.16)	0.76
Full model + energy	0.81 (0.60–1.09)	1 (ref)	0.80 (0.65–1.00)	0.88 (0.66–1.18)	0.67
	**Proximal Colon**				
Cases	36	85	128	67	
Age-adjusted model	0.82 (0.55–1.21)	1 (ref)	0.72 (0.55–0.95)	0.75 (0.55–1.04)	0.12
Full model	0.78 (0.52–1.15)	1 (ref)	0.75 (0.57–0.99)	0.79 (0.57–1.10)	0.33
Full model + energy	0.77 (0.52–1.14)	1 (ref)	0.69 (0.52–0.93)	0.66 (0.44–0.98)	0.09
	**Distal Colon**				
Cases	20	50	74	49	
Age-adjusted model	0.80 (0.47–1.34)	1 (ref)	0.72 (0.50–1.03)	0.92 (0.62–1.37)	0.90
Full model	0.83 (0.49–1.40)	1 (ref)	0.73 (0.51–1.05)	0.96 (0.64–1.45)	0.98
Full model + energy	0.83 (0.49–1.41)	1 (ref)	0.78 (0.53–1.15)	1.14 (0.69–1.88)	0.55
	**Rectal**				
Cases	26	50	98	44	
Age-adjusted model	1.07 (0.67–1.73)	1 (ref)	0.97 (0.69–1.36)	0.82 (0.55–1.23)	0.24
Full model	1.06 (0.66–1.71)	1 (ref)	0.98 (0.70–1.38)	0.84 (0.55–1.28)	0.33
Full model + energy	1.06 (0.66–1.71)	1 (ref)	0.95 (0.66–1.36)	0.78 (0.47–1.27)	0.26

^1^: whole-grain bread consumption; zero/seldom: 0 (zero/seldom), low: 14 or 34 g·day^−1^ (1–7 slices·week^−1^), medium: 100 g·day^−1^ (2–3 slices·day^−1^), high: 180 or 240 g·day^−1^ (≥4 slices·day^−1^); ^2^: age as the time scale; ^3^: age as the time scale and adjusted for body mass index, hormone replacement therapy, smoking, alcohol consumption, red meat consumption, processed meat consumption, fiber from foods other than whole-grain bread, calcium from food; ^4^: age as the time scale and adjusted for body mass index, hormone replacement therapy, smoking, alcohol consumption, red meat consumption, processed meat consumption, fiber from foods other than whole-grain bread, calcium from food, energy.

Running analyses using the full model less one variable (BMI/hormone replacement therapy/smoking/ alcohol consumption /red meat consumption/processed meat consumption/fiber from foods other than whole-grain bread/calcium from food), using the full model plus one variable (height/area of residence/period of birth/partly refined bread/folate from foods other than whole-grain bread), or using fruit, vegetables and potato consumption instead of fiber from foods other than whole-grain bread in the full model did not alter the HRs substantially for total CRC or any of the colorectal subsites. Education (*n* = 74,165) did not substantially alter the HRs for total CRC and the colorectal subsites when added to the age-adjusted and the full model, respectively. When comparing the high, the medium and the zero/seldom consumption groups with the low consumption group after excluding all CRC cases with follow-up time ≤1 year in the full model, the HRs for total CRC were 0.89 (95% CI: 0.72–1.10), 0.86 (95% CI: 0.72–1.03) and 0.88 (95% CI: 0.68–1.14), respectively. Furthermore, when performing the same comparisons for proximal colon cancer in the full model, the HRs were 0.82 (95% CI: 0.59–1.15), 0.76 (95% CI: 0.58–1.01) and 0.80 (95% CI: 0.54–1.19), respectively. Since excluding all CRC cases with follow-up time ≤1 year hardly changed the HRs for total CRC and the colorectal subsites in the three models, these CRC cases were included in the analyses. No interaction effects were found between whole-grain bread and red meat or processed meat in the full model for total CRC or any of the colorectal subsites.

## 4. Discussion

Overall, no association between whole-grain bread consumption and CRC was found in this prospective cohort study of Norwegian women. Our study does not support the findings of a meta-analysis, which indicated that there may be an inverse association between the consumption of whole grains and the risk of CRC [6]. However, when examining the colorectal subsites, whole-grain bread consumption tended to be weakly associated with risk of proximal colon cancer. Whole-grain bread was not associated with distal colon cancer or rectal cancer.

The strengths of the study include its prospective nature [35], information on many potential confounders, almost complete follow-up through linkage to national registries (the Cancer Registry of Norway has almost complete information on colon and rectal cancer and a high proportion of morphologically verified colon and rectal cancer [36]), the fact that the FFQ used in the study has been validated [27,30], and finally, that whole-grain bread consumption is generally high and varies greatly in Norway.

Nevertheless, it is important to consider several limitations. The modest number of cases and imprecise exposure assessment is of concern. We had only one measurement of dietary intake, which was obtained at the start of the follow-up, and diet, including whole-grain bread consumption, is likely to change over time [21,22,35,37]. Furthermore, CRC probably develops over decades [38], and it is uncertain whether whole-grain bread consumption may influence early or later stages of carcinogenesis [10]. Also, the classification of bread according to its content of whole grains (refined/partly refined/whole grain) may not have been clear to all participants, making it challenging to choose the correct bread type. However, such errors are most likely random among cases and the rest of the cohort and the consequence was most probably an underestimation of the true association. If many of those who reported consumption of six or more slices·day^−1^ of whole-grain bread actually consumed more than six slices of whole-grain bread day^−1^, a systematic error may have been introduced. However, we do not think this is very likely since only 2.7% of the participants reported consuming six or more slices·day^−1^ of whole-grain bread. Furthermore, another Norwegian survey found that only 2% of the women reported intake of 7–8 slices of bread day^−1^, and none reported consumption of more than 8 slices per day [22]. In general, a systematic underestimation of food intake, most probably random among the cases and the rest of the cohort, may have occurred due to the categorization in the uppermost frequency option in the questionnaire. The ability of FFQs to give precise estimates of dietary intake is a general matter of concern in observational studies. In the present study, a correlation of 0.49 between the FFQ and four 24HDRs was seen for the “Bread and cereals” category [27]. However, misclassification is not expected to be differential between persons later diagnosed and not diagnosed with CRC, and thus the consequence is most probably an underestimation of the true association. The relative validity of the NOWAC FFQ has been found to be comparable to that of FFQs used in other cohort studies [27]. The quality of our estimates is therefore considered comparable to similar studies.

The associations were estimated within a prospective cohort study, thus we can rule out the most serious concerns regarding selection and information bias; nevertheless, some issues must be considered. Over-reporting of foods that are considered healthy is a known problem [39]. Therefore, some partly-refined bread consumption may have been reported as whole-grain bread consumption [21,22]. If such a misclassification is related to the risk of developing CRC, our results may have been biased in either direction. Potential changes in food consumption prior to diagnosis may have biased the estimates in either direction (reverse causality). However, this is not very likely since excluding cases with follow-up time ≤1 year hardly changed the HRs.

Confounding is the main concern in prospective cohort studies. We used multivariable models that included established risk factors for CRC. The differences between the minimally (only age-) adjusted models and the two multivariable models were mostly minor. Nevertheless, residual confounding cannot be ruled out, for example due to measurement errors in the investigated confounders. A specific concern may be raised with regard to physical activity level. This was the only covariate with a noticeable fraction of missing values; furthermore it is well-known that it is challenging to accurately assess physical activity by questionnaires [40]. We also lacked information about family history of CRC. If persons with a genetic risk of developing CRC are more health conscious than others, this could lead to confounding. However, hereditary CRC often affects younger age groups than those included in the present study [41,42].

A previous meta-analysis indicated that there may be an inverse association between the consumption of whole grains and risk of CRC [6]. However, looking at the published cohort studies, findings are not consistent [12,13,14,15,16,17,18,19,20], and our study does not support the findings from the aforementioned meta-analysis.

It has been discussed whether the pathology and risk factors of colon cancer differ by colon subsites (proximal *versus* distal) [43,44,45,46]. A Scandinavian study (also including women from the NOWAC Study) suggested that consumption of whole grains may be differently related to proximal and distal colon cancer. The study reported multivariable adjusted incidence rate ratios (IRRs) of 0.89 (95% CI: 0.77–1.03, linearly) and 0.61 (95% CI: 0.36–1.03, fourth *versus* first quartile) when examining the association between the consumption of whole-grain products (whole-grain bread, crispbread, and breakfast cereals) and proximal colon cancer among women, and adjusted IRR of 0.98 (95% CI: 0.85–1.13, linearly) and 0.88 (95% CI: 0.52–1.50, fourth quartile *versus* first quartile) for distal colon cancer [12]. The consumption of whole grains in relation to colon subsites has also been investigated in the USA. This study (involving men and women), found multivariable adjusted RRs for proximal and distal colon cancer of 0.84 (95% CI: 0.69–1.01) and 0.85 (95% CI: 0.69–1.06), respectively when comparing the fifth with the first quintile [17]. Our significant result at the 5% level for proximal colon cancer when comparing the medium with the low consumption group may be due to chance, but it also raises the question whether the association between whole grains and CRC varies by colon subsite. A suggested mechanism behind this is that proximal and distal colon cancers may arise via different pathways [47].

Different results have been observed when examining whole grain consumption in relation to rectal cancer [12,15,18]. Our study supports the findings of a Swedish study that found no association between whole grains consumption and rectal cancer [15]. Another issue is the possible difference in the associations between CRC incidence and whole-grain wheat, rye, and oats due to differences in their content [10,48]. Wheat, followed by rye, is the most frequently sold whole-grain flour in Norway [49], so we assume that whole-grain bread consumption in our study is dominated by wheat. To our knowledge, no other studies have looked into possible associations between the different types of whole grains and the risk of CRC in the same population. However, a recent study among Norwegians, Swedes, and Danes looking into this issue found no clear picture regarding the possible different effects of wheat, rye, or oat [12].

In conclusion, overall, no association between whole-grain bread consumption and CRC was found among the women in the NOWAC Study. Our study does not support the findings of a previous meta-analysis, which indicated that there may be an inverse association between the consumption of whole-grains and the risk of CRC [6]. However, in subsite analyses, whole-grain bread consumption tended to be weakly associated with a lower risk of proximal colon cancer, and this warrants further investigation.
